# Treatment of a Large Hemorrhagic Midbrain Cavernoma Within the Silvian Aqueduct in a Five-Year-Old Girl—A Case Report

**DOI:** 10.3390/children12050564

**Published:** 2025-04-26

**Authors:** Matthias Krause, Armin-Johannes Michel, Johannes Koch, Johann Gradl, Johannes A. R. Pfaff, Christoph J. Griessenauer, Lorenz Stana-Hackenberg

**Affiliations:** 1 Department of Neurosurgery, Paracelsus Medical Private University Salzburg, 5020 Salzburg, Austria; 2 Department of Pediatric Surgery, Paracelsus Medical Private University Salzburg, 5020 Salzburg, Austria; 3 Department of Neurosurgery, Medial University Leipzig, 04103 Leipzig, Germany; 4 Department of General Pediatrics, Paracelsus Medical Private University Salzburg, 5020 Salzburg, Austrial.stana-hackenberg@salk.at (L.S.-H.); 5 Department of Radiology, Paracelsus Medical Private University Salzburg, 5020 Salzburg, Austria

**Keywords:** midbrain cavernoma, endoscopic third ventriculostomy, microsurgical resection, tectal cavernoma

## Abstract

Brain stem cavernomas are exceedingly rare in pediatric populations, with limited literature addressing their natural history, treatment guidelines, and counseling. We report the case of a 5-year-old girl presenting with acute neurological symptoms, including diplopia, gait ataxia, headache, and altered consciousness. Initial imaging revealed obstructive hydrocephalus caused by a hemorrhagic lesion near the pineal region. After emergency external ventricular drainage (EVD), most symptoms resolved except for diplopia. A subsequent MRI suggested a space-occupying hemorrhagic cyst in the tectal lamina, leading to endoscopic third ventriculostomy (ETV). During ETV, a large hemorrhagic mass at the aqueduct entrance was identified but not removed due to its fragility. Following ETV, the patient improved rapidly and was discharged. However, she was readmitted with recurrent symptoms and altered consciousness. An emergency MRI indicated a progressive hemorrhagic mass lesion compressing the midbrain, necessitating surgical intervention. The patient underwent suboccipital craniotomy using a telovelar approach. The intraoperative findings included cavernoma-like tissue within the aqueduct, which was successfully resected. Histopathology confirmed hemorrhagic and angiomatous tissue, excluding a primary brain tumor. Postoperatively, the patient showed significant, progressive neurological improvement, with mild internuclear strabism, trunk ataxia, and fatigue at the last follow-up. Six months later, a follow-up MRI and cerebral angiography showed no cavernoma remnants but identified a midbrain deep venous anomaly. This case underscores the feasibility of the microsurgical resection of midbrain cavernomas in symptomatic pediatric patients, highlighting the importance of the thorough assessment of atypical hemorrhagic midbrain lesions to exclude rare vascular malformations from differential diagnoses.

## 1. Introduction

Brain stem cavernomas are very rare entities in the pediatric population, with limited research in the literature on their natural course, guidelines for treatment, and counseling [1,2,3,4,5,6]. Most examples in the literature report adult cases of pontine cavernomas, their presentation, hemorrhage risk, and their natural course. We found only four case reports of adult patients presenting with obstructive hydrocephalus due to small cavernomas, few case reports of deep venous anomalies within the Silvian aqueduct [3,7,8,9], and only a few publications mention cases with tectal plate localization in children [6].

## 2. Case Description

**Figure 1:**
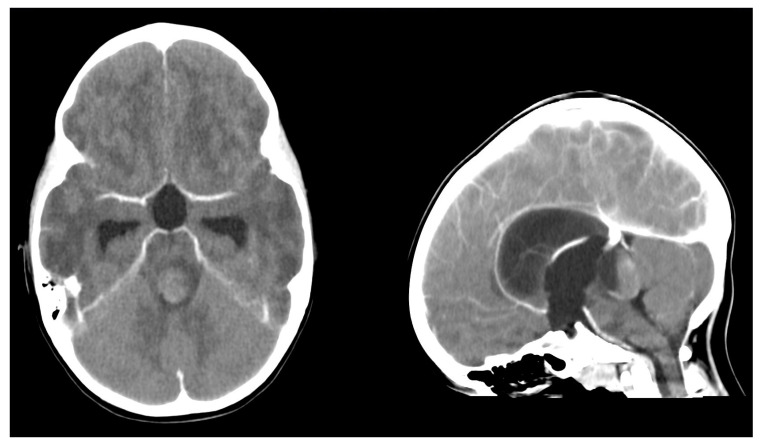
Axial and sagittal computed tomography head scan showing an acute occlusive hydrocephalus due to a mass lesion with hemorrhagic components at the midbrain occluding the aqueduct.

**Figure 2:**
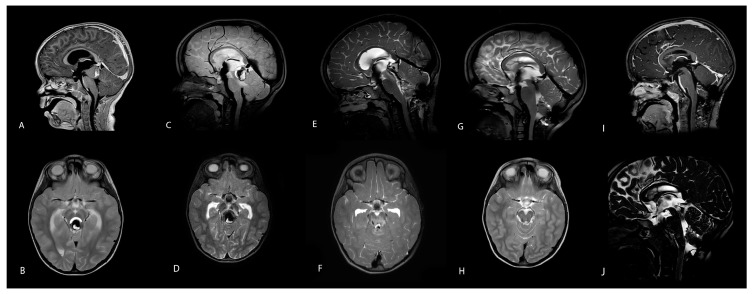
(**A**,**B**): Axial and sagittal MRI scan with a cystic and hemorrhagic lesion within the midbrain occluding the aqueduct, resulting in a compression of the midbrain. (**C**,**D**): Axial and sagittal MRI scan after endoscopic ventriculostomy (ETV) showing a flow void signal on the floor of the third ventricle and an enlarged hemorrhagic lesion with progressive midbrain compression and perifocal edema. (**E**,**F**): A postoperative axial and sagittal MRI scan demonstrated flow across the aqueduct after the removal of the lesion and residual edema. Additionally, in the axial view, deep venous anomaly (DVA) is revealed in the midbrain rostrally on the left. (**G**–**J**): Follow-up MRI 6 months after surgery showing sagittal and axial orientations in T2, CISS, and T1 with contrast disclosing the complete removal of the cavernoma, open aqueduct, and minor scar tissue remnants on the rostral lining of the aqueductal floor. The ETV flow void signal is still present.

A 5-year-old girl presented with the acute onset of neurological symptoms, including diplopia, gait ataxia, headache, and an impaired level of consciousness. A radiological examination via an emergency computer tomography (CT) scan showed obstructive hydrocephalus due to a large hemorrhagic lesion 20 × 23 × 27 mm in size in the vicinity of the pineal region (Figure 1). After the emergency placement of external ventricular drainage, all symptoms except diplopia resolved completely (Figure 2A,B). An initial magnetic resonance imaging (MRI) study led to the initial diagnosis of a non-characteristic cystic lesion (17 × 22 × 25 mm size) of the lamina tecti with a blood fluid level, with neither perilesional edema nor contrast enhancement. An MRI examination of the entire spinal column showed inconspicuous vertebral bodies and correct anatomical relationships of the neuronal structures. Endoscopic third venticulostomy (ETV) was, therefore, performed, and the lesion was inspected. This revealed a large hemorrhagic tumorous mass with a cyst at the entrance of the aqueduct. Endoscopic fenestration and irrigation of the rostral part were made, but removal was not feasible due to the vulnerability of the tissue and a noticeable intralesional clot structure in terms of increased density and firmness (Figure 2C,D).

After ETV, hydrocephalus was fully resolved, and external ventricular drainage (EVD) was weaned. Due to the rapid improvement of her neurological status, the girl was discharged for watchful waiting. However, the girl had to be readmitted after one week with recurrent symptoms of Salus–Körber–Elschnig syndrome (also known as Sylvian aqueduct syndrome and dorsal midbrain syndrome) and rapidly alternating levels of consciousness. An emergency MRI revealed a progressive mass lesion with repetitive hemorrhage expanding to and compressing the midbrain with perifocal edema without hydrocephalus (Figure 2E,F). Due to a focal deficit, open surgical resection was prompted.

The girl underwent a suboccipital craniotomy using the telovelar approach through the fourth ventricle. Intraoperatively, the lesion presented as cavernoma- or angioma-like tissue within the aqueduct. Careful microsurgical resection and coagulation of a feeding arterial vessel were performed, resulting in the complete removal of the tissue from the Silvian aqueduct and rostral midbrain. An EVD was inserted by navigation to both deflect possible re-bleeding and to regulate intraventricular pressure. A histopathological examination confirmed hemorrhagic components and hemangioma tissue, ruling out a primary brain tumor.

The postoperative course was uneventful, and the EVD was removed on the seventh day. Neurological symptoms improved significantly within three months. Residual internuclear strabism required alternating ocular occlusion therapy. The girl still suffered from trunk ataxia and fatigue at the last follow-up of 6 months, with slow improvement over time.

Due to the very unusual nature of the lesion, we performed a diagnostic cerebral angiography and MRI six months postoperatively. The MRI showed a remaining midbrain deep venous anomaly and gliotic tissue without cavernoma remnants (Figure 2G–J). The angiogram was unremarkable.

## 3. Discussion

Intracranial cavernous angiomas or cavernomas in the midbrain or brainstem are rare vascular malformations in pediatric patients, especially if they involve the tectal region. Anatomic challenges due to size, the higher risk of hemorrhage compared to adults, and the lack of substantial literature or surgery reports make them delicate surgical entities [1]. In adults, some knowledge and understanding exists regarding midbrain cavernomas, a potential subtype classification based on anatomic and neurologic clinical presentation, in addition to proposed surgical modalities for each subtype [10]. However, as in our case, clinical presentation only partially overlapped with Catapano’s published constellation of symptoms for a quadrigeminal cavernous malformation either due to this patient’s unique anatomic feature or their age [10]. The presentation of hydrocephalus and an altered state of consciousness warranted initial surgical intervention in our case. Because of anatomic challenges, their rare occurrence, and the lack of evidence in pediatric patients, an initial conservative approach is very often chosen. Since this decision has, to date, mainly been based on adult guidelines and evidence, it is not clear if this is the best strategy for a child. In the field of midbrain cavernoma in pediatric patients, risk-benefit estimations still have to be made on a case-by-case basis, and therapy recommendations need to be thoroughly individualized. In 2022, Barbarawi et al. described two children among a case series of 25 subjects with tectal cavernomas in a similar location to our patient [6]. However, Parinaud syndrome was only present in male individuals, as seen in previous reports [11,12,13]. Smaller volumes of brain stem cavernomas favor benign courses. As in the case of our patient, a large tumor mass is associated with higher morbidity due to compression, repeated hemorrhage, and brain stem dysfunction, prompting neurosurgical intervention but adequately balancing risk-benefit ratios [14]. In addition, it is unclear how pediatric patients with brain stem cavernomas should be counseled, as no pediatric studies exist to guide caregivers regarding the question of optimal therapy.

## 4. Conclusions

The microscopic resection of a midbrain cavernoma within the aqueduct is a feasible procedure in symptomatic lesions despite the delicate localization in a small child. It proved capable of alleviating the mass effect and resulted in the almost complete neurological recovery of the patient. This case demonstrates that atypical hemorrhagic lesions within the midbrain need to be carefully assessed, diagnosed, and treated to rule out rare vascular malformation as a differential diagnosis to brain tumors of that region. Despite high risks of postoperative complications and difficult decision-making, our case underscores the fact that risk stratification, a multimodal surgical approach (EDV and ETV), and highly experienced operator performance resulted in a highly favorable outcome in our young patient. Given the scarcity of pediatric midbrain cavernoma cases, future research should aim to conduct multicenter studies to develop standardized treatment protocols, especially regarding the timing of surgical intervention and the long-term monitoring of neurological outcomes. To focus expertise, children with cerebral AV malformations should be treated in centers that have both a pediatric neurosurgical team and a pediatric surgical specialist for vascular malformations. The knowledge gained on the long-term clinical course and neurological development of these children can only be applied accordingly in a central facility with specialization.

## Data Availability

The original contributions presented in this study are included in the article. Further inquiries can be directed to the corresponding author(s).

## Abbreviations

The following abbreviations are used in this manuscript:

EVD	External Ventricular Drainage
DOAJ	Endoscopic Third Ventriculostomy
MRI	Magnetic Resonance Imaging
